# Applications of Metabolomics for the Elucidation of Abiotic Stress Tolerance in Plants: A Special Focus on Osmotic Stress and Heavy Metal Toxicity

**DOI:** 10.3390/plants12020269

**Published:** 2023-01-06

**Authors:** Manamele Dannies Mashabela, Priscilla Masamba, Abidemi Paul Kappo

**Affiliations:** Department of Biochemistry, Faculty of Science, University of Johannesburg, Auckland Park Kingsway Campus, P.O. Box 524, Johannesburg 2006, South Africa

**Keywords:** abiotic stress, heavy metal toxicity, metabolomics, osmotic stress, stress tolerance

## Abstract

Plants undergo metabolic perturbations under various abiotic stress conditions; due to their sessile nature, the metabolic network of plants requires continuous reconfigurations in response to environmental stimuli to maintain homeostasis and combat stress. The comprehensive analysis of these metabolic features will thus give an overview of plant metabolic responses and strategies applied to mitigate the deleterious effects of stress conditions at a biochemical level. In recent years, the adoption of metabolomics studies has gained significant attention due to the growing technological advances in analytical biochemistry (plant metabolomics). The complexity of the plant biochemical landscape requires sophisticated, advanced analytical methods. As such, technological advancements in the field of metabolomics have been realized, aided much by the development and refinement of separatory techniques, including liquid and gas chromatography (LC and GC), often hyphenated to state-of-the-art detection instruments such as mass spectrometry (MS) or nuclear resonance magnetic (NMR) spectroscopy. Significant advances and developments in these techniques are briefly highlighted in this review. The enormous progress made thus far also comes with the dawn of the Internet of Things (IoT) and technology housed in machine learning (ML)-based computational tools for data acquisition, mining, and analysis in the 4IR era allowing for broader metabolic coverage and biological interpretation of the cellular status of plants under varying environmental conditions. Thus, scientists can paint a holistic and comprehensive roadmap and predictive models for metabolite-guided crop improvement. The current review outlines the application of metabolomics and related technological advances in elucidating plant responses to abiotic stress, mainly focusing on heavy metal toxicity and subsequent osmotic stress tolerance.

## 1. Introduction: Heavy Metal (HM) Toxicity and Implications on Crop Development and Human Health

Plants are sessile organisms in nature and thus constantly exposed to biotic and abiotic stressors. Among the various environmental factors, abiotic stressors make up most factors affecting crop growth, productivity, and yields, subsequently impacting the livelihood of individuals from underdeveloped and developing nations in the era of growing food insecurity [1,2]. In the last decade, heavy metal (HM) toxicity has become a prevalent source of concern for the agroeconomic industry owing to the threat to crop productivity. In particular, the growing practices of anthropogenic activities such as mining, industrialization, fertilizer, and pesticide applications, uncontrolled sewage disposal, and the extensive use of groundwater for irrigation have accelerated the distribution of HMs. The accumulation of these metals consequently affects soil health due to alterations in soil pH and imbalances in the ratio of essential or nonessential elements. These deleterious events further reduce available arable land for agricultural production [3]. 

HMs are a select group of metals inclusive of, but not limited to, lead (Pb), manganese (Mn), copper (Cu), nickel (Ni), cobalt (Co), cadmium (Cd), mercury (Hg), aluminium (Al) and arsenic (As), which can affect host plant performance in growth and development [4]. According to Riyazuddin and co-workers [2], adequate amounts of metal-based micro and macronutrients such as Cu, Zn, Co, Ni, Fe, Cr, Mn, I, and Se are essential for the functionality of key enzymes and the regulation of metabolic activities such as redox homeostasis, metabolism, DNA synthesis, and photosynthesis. However, excess levels of HMs exhibit toxicities that can be lethal. Plants generally tolerate unfavourable conditions when exposed for long enough (over generations). However, due to reduced mobility, or the lack thereof, plants growing in HM-contaminated soils are not spared from severe symptoms such as inadequate osmoregulation, low biomass accumulation, stunted growth, and photosynthesis, reduced yields, root browning, leaf chlorosis, altered water balance and nutrient assimilation, reduced defense capabilities or even death [5,6,7]. Essentially, plants employ strategies such as metal compartmentalization into vacuoles, sequestration through metal chelating compounds, and reduced uptake or reactive oxygen species (ROS)-activated antioxidant defense effective at moderate concentrations [8]. Additionally, HMs such as As, Ag, Hg, Cd, and Pb are of no biological significance to plants but rather are harmful, and can lead to profound health implications for humans (skin and lung cancer, urinary tract disorders, cardiovascular diseases, neurotoxicity, and diabetes) and animals upon injection [9]. Such metals can accumulate in edible parts of the plants and carry over potentially lethal health implications throughout the food chain.

Given the concerns addressed above, there is a need to understand the underlying biochemical mechanisms of HM toxicity on crops, the nature by which crops defend themselves, and the scientific tools and strategies applicable to studying these phenomena.

## 2. Combined HM Toxicity and Osmotic Stress in Plants

The impact of HMs on plant functionality extends beyond toxic accumulations to harmful levels. HMs play a crucial role in plant water relations and water availability in soils, further affecting water uptake and inducing a cascade of stress conditions. When soils are saturated with HMs, the osmotic potential in the soils becomes lower relative to the cell sap within the root system. As such, HM ions can reach concentrations sufficient to cause severe restrictions on water uptake by the plants and lead to osmotic disturbances [10]. Many studies have reported on the disruption of hormone balance, deficiency of essential nutrients, inhibition of photosynthesis, changes in photo-assimilate translocation, and alteration of water relations, which further enhance the metal-induced growth reductions in plants. However, the impacts on water relations have surprisingly received very little attention [11]. Additionally, osmotic instability and the effects thereof on plants are generally referenced as secondary to focal and extensively studied stress conditions such as drought and salinity. 

HM accumulation in soils associated with root systems affects plant water relations and osmoregulation in several ways; for instance, decreased root area was observed in *Salix caprea* following exposure to Zn and Cd combined metal stress [12]. Other reported anomalies include reduced root elongation, impaired secondary growth, and reduced root hair surface area because of Ag, Ni, and Pb exposure [13,14,15]. Taken into consideration, these metal-induced structural alterations result in inadequate root-soil contact and thus reduced capacity of water uptake by the plant, combined with the accumulation of the HMs in the soil, which drives the concentration of metal ions to exceed the threshold, an osmotic disturbance due to lowered osmotic potential becomes inevitable. Cheng et al. [16] observed combined heavy metal contamination (400, 400, and 600 mg kg^−1^ DW of Cu, Pb, and Zn, respectively) in *Kandelia obovata*; the findings showed that the mangrove plant could tolerate elevated levels of HMs but only with a reduced osmotic adjustment which was functional for a limited time. A study on combined osmotic and cadmium (Cd^2+^) stress on *Brachypodium* seedling roots found that the two factors were responsible for the inhibition of seedling growth and led to significant phenotypic and physiological changes, including decreased relative water content, primary root length, and plant height [17]. The authors concluded that combined osmotic and Cd^2+^ stresses had more severe effects on seedling growth than independent stress, thus revealing a correlation between HM toxicity and limited osmotic regulation by the host plant.

Osmotic stress can be catastrophic if it continues to receive less attention than deserved, mainly because the advent of osmotic stress in plants potentiates the effect of other stressors on plants, further exacerbating damage to cellular structures and functionality. For instance, an apparent osmotic imbalance in plants is followed by fundamental oxidative damage, which results from an altered electron transport chain and reduced mitochondrial respiration [17]. The results are overproduction of harmful free radicals and reactive oxygen species (ROS) [18], which consequently cause damage to transpiration, photosynthetic and DNA/RNA synthesis machinery leading to retarded growth and productivity of plants or even death [18,19,20]. Additionally, HM toxicity drives similar effects on plants through the overproduction of ROS, leading to oxidative stress; this is characterised by the loss of cellular membrane structure and function due to lipid peroxidation [20]. Furthermore, an accumulation of HMs such as Pb, Cd, and Hg has been reported to increase the production of ROS as hydroxyl radical (OH), Superoxide radical (O_2_^−^), or hydrogen peroxide (H_2_O_2_) in plants which inherently compromise the plant’s antioxidant defence [9,21,22]. Moreover, HMs cannot undergo degradation or modification as toxic organic compounds and thus persist in cells and continuously interfere with cellular homeostatic pathways [4,23]. Therefore, the potential damage incurred in plant exposure to HM and osmotic stress is evident. 

### 2.1. Plant Response and Adaptation for Tolerance to HM Toxicity and Osmotic Stress

**Figure 1:**
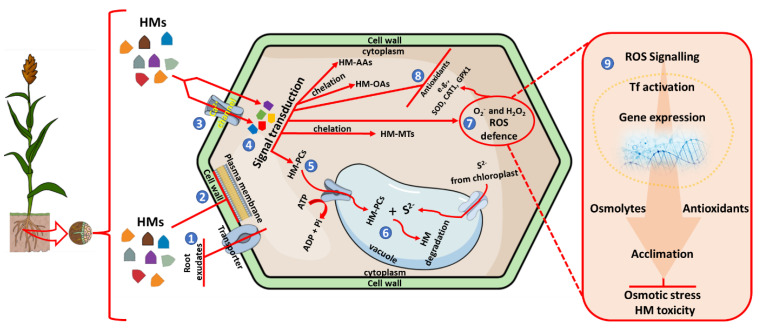
Schematic representation of plants’ response to heavy metals and induced osmotic stress. Plants growing in HM-contaminated soils can prevent HM uptake by (1) releasing root exudates that form insoluble complexes with metal ions, and (2) reduction of metal influx across the plasma membrane. Metal ions influx can still occur through the Ca^2+^ channels and the plasma membrane into the plant cell (3), MAPK-mediated signal transduction (4), occurs to induce the expression of metal chelating ligands in the cytosol to form complexes such as HM-AAs (amino acids), HM-OAs (organic acids), HM-MTs (metallothioneins) and HM-PCs (phytochelatins). HM-PC complexes are transported through the tonoplast into the vacuole by ATP-binding-cassette and V-ATPase transporter (5), which are further complex with sulfides from the chloroplast for degradation (6). The perception of HMs is amplified by the signalling transduction to induce ROS defence and oxidative stress (7), this leads to the activation of antioxidants (8) and (9) to combat oxidative damage, osmotic stress, and HM toxicity.

Plants are limited by their sessile nature from escaping particularly unfavourable changes in environmental conditions; additionally, each cycle of exposure to HM and osmotic stress leads to further physiological and biochemical alterations that are deleterious to the host. Plants have thus developed adaptive strategies to combat and cope with the harmful consequences of singular or combined HM toxicity and osmotic stress as illustrated in Figure 1. This response is undoubtedly evoked following a systematic approach to modulate molecular and biochemical mechanisms of stress response defined by (i) the perception of environmental stimuli, (ii) intracellular signal transduction to relay the specific message, and (iii) a cascade of gene activation mechanisms and reprogrammed metabolic processes in response to the perceived stimuli to counteract the effects of the environmental stressor [6]. Due to conditions such as HM toxicity and osmotic stress, physiological changes in plants succeed in biochemical alterations and are generally visible at the dire stages of exposure. As such, monitoring the onset of intracellular responses such as oxidative stress or signalling cascades at the genomic, transcriptomic, proteomic, and even metabolic levels could be a proactive measure to detect stress-specific signal transduction. Reports have shown that early signs of metal toxicity occur similarly to other environmental stresses, such as osmotic or drought stress, oxidative stress, nutrient imbalance, and altered photosynthetic and developmental parameters [10,24]. As such, an apparent interconnectedness of regulatory networks has thus been established, which one could call a standardised (nonstress-specific) plant response to external stimuli, which results in the activation of various genes and transcriptomic regulatory factors. In the context of HM toxicity and osmotic stress, genes responsible for metal chelators and ion transporters are thrust into activity from varying pathways. Over the years, biologists have deciphered the mechanisms involved in plant response to abiotic stress conditions with great attention to common stresses such as drought and salinity. These mechanisms are briefly discussed below, focusing on HM toxicity and osmotic stress response. It is important to note that these mechanisms are primarily made up of interconnected regulatory networks, as previously mentioned, and are thus similar in responding to various environmental stressors. Moreover, HM toxicity triggers osmotic imbalances in plants; as such, the response mechanisms associated with osmotic stress can be attributed mainly to plant response to HM toxicity.

Mitogen-activated protein kinases (MAPKs) are a set of regulatory kinase proteins involved in directing cellular responses to a diverse array of stimuli by relaying extracellular signals to intracellular responses, thus regulating cell proliferation, differentiation, motility, and survival [25]. MAPKs are some of the most important and highly conserved regulatory signalling molecules active in plant response and plants’ general developmental stages, including many cellular processes [24]. MAPK-mediated plant response is activated by specific metal ligands and ROS molecules produced during metal stress; the perception of these stimuli triggers a MAPK signalling cascade and signalling transduction pathways [6,24]. This cascade consists of three kinases (MAPKs, MAPKKs, and MAPKKKs) activated by a relay phosphorylation process that ultimately phosphorylates downstream target substrates such as transcription factors [24,25]. 

Many studies characterizing MAPK-mediated plant response to HM toxicity or osmotic stress have been on model plants, such as *Arabidopsis*. For instance, Ye et al. [26] reported MPK6 activation from Cd exposure in *Arabidopsis* plants, which triggered caspase-3-like activation, and led to a defence mechanism for stress response through programmed cell death (PCD). The role of MPK3 in metal response was demonstrated in *Arabidopsis* following exposure to copper (Cu) and Cd [27]. In a study investigating the effects of MT on antioxidant capacity in naked oat seedlings under drought stress, Gao and colleagues [28] found the up-regulation in the expression rates of MAPKs (*Asmap1* and *Aspk11*) along with transcription factor (TFs) (TF) genes *WRKY1*, *DREB2*, and *MYB* triggering the regulation of downstream antioxidant and drought stress response-related genes to enhance plant tolerance and thus the alleviation of osmotic stress. The expression of MAPKs also enhanced superoxide dismutase (SOD), peroxidase (POD), catalase (CAT), and ascorbate peroxidase (APX) activities in the leaves of naked oat seedlings under drought stress. Few studies have elucidated the MAPK-mediated response mechanisms of plants to metal toxicity and osmotic stress, particularly in the last decade. However, studies on fish (carp) [29], fungus (*Trichoderma atroviride*) susceptible to osmotic stress, oxidative stress, and Cd toxicity [30], yeast responding to Cd [31] and erythrocytes following osmotic shock [32] have proven the activation of MAPKs and MAPK-activated stress response pathways and several transcription factors downstream of MAPKs due HM and osmotic stress. All these studies imply the involvement of MAPK cascades in stress responses. Considering reports highlighting the excessive contamination of the environment through industrial discharge of heavy metals, special attention needs to be paid to plant response mechanisms of these abiotic stresses.

Other response mechanisms have been shown to alert plants of HM and osmotic stress conditions inclusive of calcium signalling, in which a rapid increase in cytosolic Ca^2+^ ([Ca^2+^] cyt) follows the perception of a stimulus from nanomolar concentrations under normal physiological conditions to gradients of numerous magnitudes and triggering complex signalling transduction and metabolic pathways [33]. The perception of an environmental stimulus induces [Ca^2+^] cyt fluctuations which trigger calcium sensors such as calcium-dependent protein kinase (CDPK) to relay information that activates downstream targets through signal transduction [34]. In plants responding to HM exposure, calcium (Ca) has been shown to reduce Cd toxicity due to similarities in Cd and Ca physiochemical properties, transport channels, and intracellular binding sites [6]. When plants perceive a Cd stimulus, Ca sensors induce an increase in intracellular Ca^2+^ as an adaptive measure to mitigate the toxic effects of the heavy metal [24]. The exogenous application of Ca^2+^ was found to reduce toxicity and increase tolerance of *Brassica juncea* to Cd stress leading to improved seedling growth [35]. A study by Zhao et al. [36] further suggested crosstalk between signalling pathways in *Exophiala pisciphila* plants responding to Cd through Ca^2+^-mediated maintenance of homeostasis. In the roots of *Arabidopsis* seedlings, osmotic stress stimulus triggers a rapid increase of [Ca^2+^] cyt suggesting cytosolic calcium to be involved in the osmotic stress response [37]. Additionally, plants respond to osmotic stress by activating Ca^2+^ signalling, accumulating the stress hormone abscisic acid (ABA), reprogramming gene expression, altering growth, and improving stress tolerance [38].

Plant hormones coordinate adaptive changes in cellular osmotic regulation by regulating various molecular events under specified environmental conditions. ABA is a plant stress-signalling hormone involved in plant abiotic stress response [39]. Under osmotic drought conditions, HM toxicity, and high salinity, ABA induces short-term defence responses such as stomatal closure to maintain homeostasis, generally followed by long-term plant growth regulation through stress-responsive expression genes [40]. ABA biosynthetic genes such as the *ZEAXANTHIN EPOXIDASE* gene (*ZEP*), the *ALDEHYDE OXIDASE* gene (AAO3), a *9-CIS-EPOXYCAROTENOID DIOXYGENASE* gene (NCED3), and the *MOLYBDENUM COFACTOR SULFURASE* genes are up-regulated upon the occurrence of osmotic stress [40], leading to an increased endogenous level of ABA and the subsequent ABA-dependant gene expression [39]. In addition, auxins, jasmonates, cytokinin, and ethylene also play crucial roles in the developmental changes effected by osmotic stress. For instance, osmotic stress stimuli modulate auxin responses by mediating auxin biosynthesis (*YUC*, *TAA1*), transport (*PIN*), and perception (*TIR/AFB*, *Aux/IAA*) [41].

On the other hand, jasmonates (JA) have been shown to induce tolerance to osmotic stress in rice, evidenced by an increased JA content of wild type (WT) compared to a mutant disrupted by JA biosynthesis [42]. These phytohormones also modulate lateral root formation and patterning and have thus been suggested to be involved in remodelling root systems and architecture in response to HM stress [24]. The ability of plants to limit the deleterious effects of HM further shields said host plants from exposure to osmotic stress; plants thus apply other mechanisms to combat HM toxicity, such as the production of ROS limited from exceeding the toxic threshold to help the plant acclimatise to the environmental stress conditions. Plants also implement antioxidant defence systems to manage ROS production, translocation, and activity through ROS scavenging; these strategies have been well documented in detail as classical plant defence mechanisms. However, recently there has been significant interest in melatonin, a pleiotropic signalling molecule reported to be at the centre of plant defence response as a universal regulator of the plant response strategies discussed above [43].

Several studies have reported the endogenous accumulation of melatonin (*N*-acetyl-5-methoxytryptamine, MT) in plants responding to HM toxicity. It was found that MT reduces Cd uptake and mitigates toxicity in plants; MT supplements significantly increased Cd tolerance and reduced Cd content in leaves of tomato (*Solanum lycopersicum* L. cv. Hezuo 903) as evidenced by decreased growth inhibition, photoinhibition, and electrolyte leakage [43]. Additionally, MT was found to confer tolerance against Cd stress in transgenic *Arabidopsis* plants through the expression of alfalfa serotonin *N*-acetyltransferase (*SNAT*); MT-treated plants also showed the up-regulation of *PDR8* and *HMA4,* which led to enhanced Cd tolerance seen through the decreased Cd accumulation in the re-establishment of microRNAs-mediated redox homeostasis [44]. Similar observations were made from studies on the root tissues of *Hordeum vulgare* (barley) [45] and *Lupinus albus* (lupin) [46], as well as leaves of *Nicotiana tabacum* (tobacco) [47] and tomato [48] as reviewed by Hoque et al. [49]. In all these studies, MT biosynthesis was flagged as one of the primary regulatory mechanisms as a biostimulator for MT-mediated alleviation of HM toxicity by regulating the expression of target genes and the modulation of heavy metal transporters. Gu et al. [44], further suggested MT as an elicitor of *miRNA*-mediated ROS homeostasis in plants responding to metal stress. 

According to Lee and Back [48], plants respond to HM toxicity and other osmotic stress inducers, such as drought and salinity, by inducing MT biosynthesis for resistance and tolerance. This pleiotropic signalling molecule fosters plant tolerance to HM toxicity by up-regulating stress-related genes, thus stimulating plants’ ROS scavenging and antioxidant capacity to improve various physiological, morphological, and biochemical features that lead to plant growth and growth development [49]. Furthermore, the role of MT in osmotic stress management has been gaining attention in recent years. Reports have suggested that MT reduces the inhibitory effect of osmotic stress on the antioxidant properties of plants such as soybean [50], and improves stress tolerance by mitigating osmotic and oxidative stress in maize seedlings [51]. Melatonin was reported to improve growth and yields in the lemon verbena plant by regulating mineral homeostasis and osmolyte accumulation [52]. However, the biochemical avenues by which MT participates in osmotic stress tolerance remain elusive; most recently, scientists have been exploring plants’ underlying mechanisms of MT-mediated osmotic stress response. For instance, Wang and colleagues [53] found that the phytomelatonin receptor CAND2/PMTR1 plays a crucial role in MT-guided plant response to osmotic stress. 

On the other hand, glutathione (GHS) is the most abundant low molecular weight, ROS scavenging, non-enzymatic antioxidant synthesized in plant cells [54]. GHS functions in mitigating osmotic stress, oxidative damage, alleviation of xenobiotic electrophiles (GHS-conjugation), and the maintenance of redox homeostasis in cells through the 1-ascorbate-glutathione (GHS-AsA) cycle [55], and also an observed tolerance in Cd-treated wheat (*Triticum aestivum* L.) by Qin et al. [56]. A study by Khan et al. [57] revealed that melatonin priming significantly induced the accumulation of GSH and AsA in rapeseed seedlings responding to induced drought stress and the subsequent ROS accumulation caused by osmotic imbalance. The findings thus suggested the ameliorative effects of melatonin priming to come through enzymatic antioxidant activity via osmotic adjustment and increased osmoprotectants. Still, most importantly, MT was discovered to enhance the non-enzymatic GSH-AsA cycle in plants. Furthermore, MT activates antioxidative enzymes such as APX, CAT, SOD, peroxide dismutase, peroxidase, dehydroascorbate reductase (DHAR), and GSH reductase during osmotic stress [58,59,60]. 

Moreover, reports have shown that the role of MT in plant response to both HM toxicity and osmotic stress and related abiotic stress conditions drives the primary response mechanisms of plants to environmental stress. For instance, mitogen-activated protein kinases (MAPKs) are a set of regulatory kinase proteins involved in directing cellular responses to a diverse array of stimuli by relaying extracellular signals to intracellular responses, thus regulating cell proliferation, differentiation, motility, and survival [25]. According to the authors, the findings present a new avenue for MT-mediated plant stress response. From the studies summarised above, MT has been shown to play a crucial role in plant HM toxicity and osmotic stress response by modulating the primary regulatory response mechanism in host plants. The said regulatory mechanisms have been well documented over the years; however, the role of MT in these processes is yet to be fully understood and thus presents an opportunity for advanced research into the exogenous applications of MT in HM toxicity and osmotic stress tolerance in plants. In any case, abiotic stress response mechanisms are defined through metabolic pathways and metabolite perturbations. Monitoring these changes could give insight into strategies for maintaining metabolic homeostasis and allows the production of defence-related compounds that aid in stress tolerance [34].

### 2.2. The Scope of Metabolomics in Plant Research: Exemplary Classical Standards and Modern Developments

Plants are essentially defenceless pertaining to physical mobility to escape changes in environmental conditions such as abiotic stress factors and are thus constantly exposed to these factors, which ultimately impact negatively on plant growth and development, as well as crop productivity and yields as previously highlighted. Therefore, there is a need to comprehensively elucidate the biochemical nature of plant responses to these abiotic stress factors to understand and subsequently predict plant metabolism under such conditions. These actionable insights should thus contribute to translational research for application in developing plants with enhanced resilience and productivity and support strategies that promote plant growth under abiotic stress conditions [61]. Section 3 above casts light on different plant response strategies and (HM toxicity and osmotic) stress signalling mechanisms uncovered over the years, while bringing much-needed attention to recent discoveries worthy of thorough investigations. However, these previously elucidated mechanisms and how they have been understood to encompass the broader spectrum of plant responses to environmental conditions only serve as the gateway to more questions and the need to understand these mechanisms beyond the current models holistically. This is to say, explorative techniques with much more robust predictive models to describe changes in the central dogma pipeline-gene expression to metabolite reprogramming-resulting from sensing mechanisms and signal transduction are yet to be fully exploited. 

**Figure 2:**
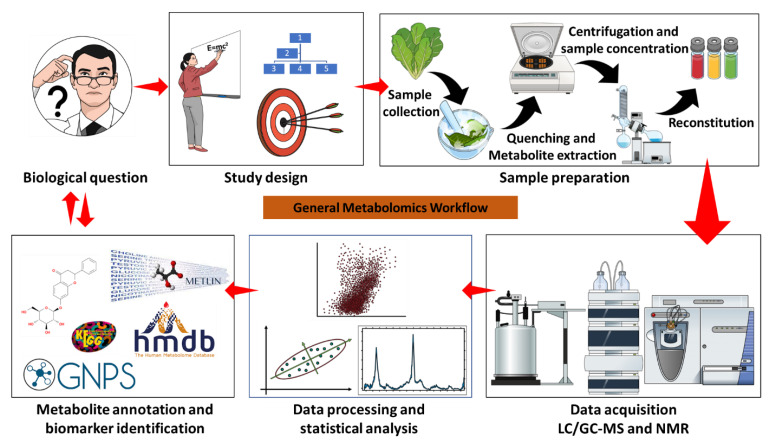
General metabolomics workflow. A typical metabolomics study is driven by a biological question, e.g., the elucidation of metabolic reprogramming in plants responding to abiotic stress. A study design determines the experimental parameters to source sample material, a suitable methodology for sample preparation, and the type of instrumentation to be used based on the research question and the aim of the study. Sample preparation can be different for NMR, LC-MS, and GC-MS, whereas data acquisition can be instrument-specific based on the experimental design and the objectives of the study, for instance, GC-MS is specific for the analysis of volatile compounds, and derivatization is required for non-volatiles. NMR is used for the elucidation of molecular and structural properties of compounds, while MS is used to determine the identities of unknown compounds using their molecular weight/formula to reveal structural and chemical properties. Specialised software (vendor-specific or free platforms) is used for data mining, processing, statistical analysis, and compound annotation and identification of biomarkers.

As such, the biochemical rationale of signal transduction and the roles of metabolic features involved remain elusive; however, the metabolite-guided abiotic stress response in the plant has gained much attention in recent in vivo and in vitro studies using metabolomics and transcriptomic technologies suggest stabilizing effects of metabolites in stress response [62,63,64]. Many such studies have applied omics sciences (genomics, transcriptomics, proteomics, and metabolomics) in integrated and independent approaches to unravel the metabolism of plants under specified abiotic stress conditions. Additionally, improved bio- and cheminformatics tools have been used to understand complex processes associated with elucidating spatial and temporal production of the bioactive compounds involved in abiotic stress perception, signalling, and response [64]. This section of the review thus focuses on the potential applications of metabolomics and the still relevant old, as well as the exciting new, related bio-/cheminformatic tools (Figure 2) to elucidate plant response to abiotic stress, particularly in HM toxicity and osmotic stress conditions. Firstly, the current advances in metabolomics technologies will be briefly discussed, followed by insights into the application of metabolomics in plant science.

### 2.3. Recent Advances in Techniques and Technologies for Metabolomics Studies

The science of metabolomics is mainly concerned with the quantitative and qualitative elucidation of the biochemical space of biological organisms; it entails the comprehensive analytical analysis of low molecular-weight compounds called metabolites (1500–1800 Da) present in the biological specimen under specified conditions [65,66,67]. The resulting snapshot of the metabolic profile, also called the “metabolome”, from the source specimen (plants) provides emphasis on the correlation between the observed metabolic concentrations, and the presence or absence thereof, with the physiological condition and cellular state of the organism and its current state of interaction with the surrounding environment [67,68]. As such, metabolites are considered the end products of cellular function, and variations in their levels can represent the whole plant’s state. The plant metabolome is enormous and highly complex compared to other organisms, consisting mainly of primary and specialised secondary metabolites, each responsible for a set of crucial metabolic functions ranging from plant growth and development—primary metabolism—to plant response to a/biotic stress and survival—secondary metabolism [66]. Therefore, integrated metabolomics approaches are required to deconvolute and elucidate the metabolic configurations, reprogramming, and regulation under varying environmental conditions, particularly in a/biotic stress response. Hence, employing advanced and appropriate analytical tools is crucial. In the current state of metabolomics studies, integrated separatory and detection technologies are used, including non-destructive nuclear resonance spectroscopy (NMR) and mass spectrometry (MS)-based methods, often in hyphenation with liquid- and gas chromatography (L/GC) or capillary electrophoresis [66,67].

#### 2.3.1. Advances in NMR-Based Analytical Techniques

In the broader spectrum of metabolomics applications, NMR methods have been relegated in favour of MS-based analytical platforms due to their low sensitivity, low resolving power, and much lower dynamic range resulting in limited metabolic coverage [69]. In recent years, significant improvements have been made in NMR-base metabolomics studies; these include the development of cryogenic probes which can handle analytical conditions at −253 °C, and multidimensional NMR, which determines the 3D structures of organic and biomolecules, leading to increased sensitivity, resolution, and reduced acquisition times [61,69]. Further advances are being made in NMR instrumentation, such as permanent magnet (neodymium-boron-iron), benchtop NMR instruments that offer excellent resolution (approaching 1 Hz), fast FT-based acquisition, multiple nuclei detection, very simple user interfaces, and a range of multidimensional spectral acquisition options for chemical identification and quantification [70]. Additionally, recent advances in magnet technology have led to the development of maintenance-free cryostats function without liquid nitrogen and only automatically recycled liquid helium. Furthermore, probe technology has improved significantly, offering cryogenically cooled probes (cryoprobes) with increased signal sensitivity and reduced sample size [71].

The development of 2D NMR improved the peak overlap or peak position biases problems in 1D NMR caused by many ^1^H peaks per metabolite [70]. Two-dimensional NMR (2D NMR) increases sensitivity and enhances resolution by untangling overlapping NMR signals [70]. Essentially, multidimensional NMR offers more resolving power through optimized approaches such as 2D ^1^H, ^13^C-heteronuclear single quantum correlation spectroscopy (HSQC), heteronuclear multiple bond correlation (HMBC), correlation spectroscopy (COSY), and total correlation spectroscopy (TOCSY) [70,71]. In addition, the selectivity and resolution of NMR analysis have been improved significantly by the application of isotope-labelled NMR. ^13^C, ^15^N, ^2^H, and ^31^P-based isotope labelling has provided the opportunity to increase the detection limit of low-concentration metabolites with high accuracy; labelled probes allow the ability to trace specified metabolic pathways and networks such as those involved in stress tolerance and adaptation [70,71]. 

#### 2.3.2. Advances in MS-Based Analytical Techniques

MS allows the detection of hundreds to thousands of metabolites in a single run, often with high sensitivity compared to NMR. Metabolites are detected using their mass-to-charge ratio corresponding to their molecular ions, making MS one of the most powerful techniques for the structural elucidation of metabolites. Automation in mass spectrometric analysis has been the central focus in analytical laboratories at the dawn of developed microcomputer technologies [72]. From sample preparation, injection, data acquisition, mining, and processing to biological interpretation, automation can improve precision and accuracy to minimise human error while also increasing efficiency and selectivity with high throughput systems [69,72]. For instance, manual sample injection into GC-MS analytical instruments requires precise injection timing and operation, which rely on the analyst’s skills. The margin of error becomes greater with larger quantities of samples to be analysed. However, the introduction of autosamplers has provided precise sample delivery and sample aliquoting to analytical instruments in the long run; additionally, injection parameters such as speed, depth of sampling, and wash cycle frequency can be pre-defined by the user for consistent operation without constant supervision [73]. LC- and GC-MS instruments can also be equipped with cartesian coordinate robots using 3D (XYZ) space movements to provide flexible platforms for numerous applications, particularly in sample preparation [73].

Metabolomics studies are further complicated by the diverse metabolite composition and complexity of the plant metabolome, including polar, semipolar, and nonpolar metabolites, volatiles, and a plethora of isomers which bring about complications with separations, selectivity, and resolution. Outstanding contributions have been made for improved separation, resolution, and the identification of individual metabolites with the application of two-dimensional workflows such as 2D-GC (GCxGC) and 2D-LC-MS [74]. General instrumentation of 2D-chromatography involves using an autosampler with binary or quaternary pumps, switching valves, two-column compartments often hyphenated to two diode array detectors, or an MS detection system [75]. In 2D chromatographic separation, the unresolved analytes from the ^1^D GC/LC column are transferred to the ^2^D column for further separation based on the differences in the resolving power or selectivity of the two dimensions, thus enhancing resolution and peak capacity [76]. These improvements have therefore allowed for better detection limits, higher quantification, and identification capacity when coupled to MS systems. This could improve metabolite coverage and elucidate the plant’s metabolic and cellular state under varying environmental conditions. 

Additionally, metabolomics analysis relies on data from extracted metabolites of biological specimens, with successful application in monitoring plant metabolic reprogramming. However, spatial localisation of metabolites remains elusive, a critically important aspect of metabolic evaluation and tracing of metabolites in and between cellular compartments such as organelles, cells, tissues, or organs. Alexandrov [77] explains the concept of spatial metabolomics as an emerging field of omics research that has enabled localising metabolites from cells, tissue, organs, and organisms. Spatial metabolomics is aided by allowing technology-imaging mass spectrometry (MSI)-to achieve robust metabolite coverage with excellent sensitivity [77,78]. Additional improvements to the metabolomics workflow further include the application of microtechnology (e.g., lab-on-a-chip, microfluidics) coupled with electrospray ionization or matrix-assisted laser desorption/ionization mass spectrometry (ESI/MALDI-MS) can help increase throughput, sensitivity, reproducibility, and data reliability as detailed in Tinte et al. [69], Elpa et al. [73], and Siegel et al. [78]. The significant improvements to metabolomics analysis are shaped by miniaturisation and computer-aided automation, machine/deep learning (M/DL) software, and algorithm-based data mining, processing, and optimisation. For instance, M/DL algorithms and artificial neural networks (ANNs) offer efficient modelling and integration of multi-omics data (genomics, transcriptomics, proteomics, and metabolomics). These computational tools have recently been explored in biomarker discovery from natural products [79], and metabolomics-guided taxonomical classification of different *Camelina sativa* varieties [80]. Complete adoption of the advancements as mentioned earlier in plant metabolomics still requires a consensus backed by proven usability; these tools would bring about improvement on the conventional multivariate data analytical (MVDA) and statistical tools such as principal component analysis (PCAs), partial least squares discriminant analysis (PLS-DAs) and binary classifiers such as orthogonal partial least squares discriminant analysis (OPLS-DA). Other computer-aided advances to manual aspects of the metabolomics workflow, such as metabolite annotation, are further discussed by Tinte et al. [69]. The review highlights potential resolutions to large-scale metabolite annotation, the bottleneck in metabolomics studies, through the introduction of web-based ecosystems such as artificial intelligence (AI)-aided 4IR technologies for s high reliable/quality data acquisition, cloud computing, and machine learning algorithms that facilitate rapid searching in library databases including molecular networking tools from the Global Natural Product Social Molecular Networking (GNPS) site and related sites like MS2LDA for data mining based on spectral and structural similarities from big metabolomics data sets. GNPS is a freely accessible web-based platform for tandem MS (MS/MS) data analysis, curation, and dissemination [81,82], coupled with MS2LDA, which enables the extraction of substructural diversity within classes of metabolites from complex datasets to provide meaningful biochemical interpretations [82], have revolutionised metabolomics data analysis as an additional avenue to other well-established platforms such as MetaboAnalyst and XCMS-to name but a few-in the 4IR era, particularly in plant metabolomics research for plant resilience to a/biotic stress.

## 3. Metabolomics for the Elucidation of Abiotic Stress in Plants: HM Toxicity and Osmotic Stress

The exposure of plants to abiotic stresses negatively affects physiological processes such as growth, development, productivity, and yields. A plant’s cellular processes and metabolic status change as the host activates complex cellular responses, including gene interactions and crosstalk with different molecular pathways and networks to combat various environmental conditions. The full extent of the functional mechanisms of these metabolic networks and associated metabolic markers are yet to be fully elucidated and thus require further investigation, more so concerning plant interactions with generally neglected stress conditions such as heavy metals and induced osmotic stress leading to metabolic perturbations. The primary goal of studying metabolic changes in plants is to elucidate metabolic features, also called metabolic biomarkers, that function to re-establish cellular homeostasis and regular metabolic activity in stressed plants to mediate stress response and tolerance [66]. The plant metabolome, the complete set of low molecular metabolites produced by plant cells under certain conditions at a set time, modulates processes at macro molecular levels; such processes can be observed with the application of several analytical techniques [64]. Of such methods in existence, metabolomics analysis has revolutionised the phytochemical landscape by facilitating the global profiling and characterisation of functional primary and specialised secondary metabolites in plants expressed under varying environmental conditions, particularly in a/biotic stress response, resistance, and tolerance [64,83]. Over the years, metabolomics-based phytochemistry has enabled a thorough understanding of metabolic pathways, metabolite biosynthesis, and bioactivity under diverse environmental conditions. It continues to gain momentum as a versatile instrument in any (phyto) chemist’s toolbox. This section of the review illustrates the applications of metabolomics in abiotic stress tolerance in plants with a special focus on HM toxicity and osmotic stress; such studies have been rare in the last decade, with relative prevalence in the noughties (2000–2010). However, with the rise in anthropogenic activities leading to heavy environmental contaminations affecting crop productivity and yields, applying new methodologies such as metabolomics could be the gateway to mediating the deleterious effects of these heavy pollutants.

### 3.1. Applied Metabolomics in the Elucidation of Plant Response to HM Toxicity

Plants growing in HM-contaminated soils are discernible through symptoms such as chlorosis, stunted growth, halted macro/micronutrients, water translocation from roots to shoots, and altered physiological processes, including photosynthesis and water relation [84]. Assessing such effects and the counter mechanisms of plants would help devise predictive measures to improve plant tolerance against HM toxicity. Luo et al. [85] collected *Whitmania pigra*, called Mahuang (MH), exposed to Pb for analysis on an ultra-performance quadrupole time-of-flight MS (UPLC-Q/TOF-MS). Metabolomics results showed significant changes in 24 metabolites from the Chinese medicinal plants responding to Pd exposure. Altered metabolites included lipids, nucleotides, and dipeptides, facilitating glycerophospholipid metabolism, sphingolipid metabolism, and nucleotide metabolism. Most recently, UHPLC-QTOF-MS profiling of tobacco plants exposed to Cd toxicity revealed significant alterations in 150 and 76 metabolites from roots and leaves under Cd stress, respectively [86]. The study showed differential regulation of metabolites associated with the biosynthesis of amino acids, nicotinate and nicotinamide metabolism, arginine and proline metabolism, and flavonoid biosynthesis. Proline has previously been shown to ameliorate photosynthetic attributes, nutrient uptake, and oxidative stress in pigeon pea plants (*Cajanus cajan* L.) under Cd stress [87]. At the same time, flavonoid-rich seedlings of buckwheat cultivars were found to be more resistant to Pb^2+^ and Cd^2+^ than seedlings with lower flavonoid content [88]. Similar observations were made by Mashabela et al. [7] when a UHPLC-QTOF-MS-based metabolic characterisation of wheat cultivars (*Triticum aestivum* L.) revealed higher levels of flavonoids in aluminium (Al^3+^) resistant varieties.

The response of tomato plants (*Solanum lycopersicum*) to long-term Cd exposure was evaluated using ^1^H NMR and HPLC-PDA [89]. Absolute metabolite quantification showed significant increases in soluble carbohydrates and sugar content in mature leaves of tomato plants exposed to 20 µM of Cd, suggested to be due to Cd interference in carbohydrate metabolism, these findings correlated with the observed increase in sucrose content compared to decreased glucose and fructose levels in the same leaves suggested to be due to Cd-induced inhibition of invertase activity [90,91]. Additionally, the study reported increased levels of α-tocopherol and ascorbate in Cd-treated leaves; both metabolites are involved in plant antioxidant defence to prevent lipid peroxidation by scavenging lipid peroxyl radicals in thylakoid membranes, thus promoting plant stress tolerance. 

Roots are the primary site of contact with metals in HM-contaminated soils, facilitating HM bioavailability and uptake by HM accumulator plants for bioremediation [6]; however, these activities are harmful to non-HM accumulators and can be mitigated by plant-derived metabolites called root exudates, thus promoting plant tolerance to HM toxicity [92]. Elucidation of root-mediated resistance strategies is crucial to understanding plant resistance mechanisms and survival strategies in HM-contaminated environments. Metabolomics analysis was used to evaluate the response of rice cultivars (*Oryza sativa* L.) under Cd stress conditions [93]. Untargeted UPLC-ESI-Q-TOF-MS-based metabolic profiling analysis of Cd-treated cultivars revealed the upregulation of lipids and fatty acids in the low Cd accumulating cultivar (TY816) compared to the high Cd accumulator (JY841). According to Liu and colleagues, HM toxicity induces the release of fatty acids and lipids in varying quantities from host plants as a cellular membrane-mediated defence mechanism against Cd stress. Here, the perception of Cd induces oxidative stress, which triggers the desaturation of membrane lipids to increase fatty acids exudation from roots into the soil (rhizosphere); membrane lipid desaturation further helps with resistance to membrane damage [93]. 

In another study, the exudation of low molecular weight organic acids (LMWOAs) was demonstrated as an essential response mechanism of plants to phytotoxicity caused by heavy metals [94]. An HPLC-PDA (photodiodes detector) analysis of root exudates from *Poa annua* L. (Poaceae; PO), *Medicago polymorpha* L. (Leguminosae; ME), and *Malva sylvestris* L. (Malvaceae; MA) plants revealed increased exudations of oxalic acid, malic acid, citric acid and fumaric acid following plant treatment with Cd, Cu (copper) and Zn (zinc). According to the authors, plants respond to HM toxicity by releasing LMWOAs as an exclusionary mechanism to reduce HM uptake, allowing plant growth in high HM contamination. Organic acids such as oxalate, citrate, malate, and fumarate have the metal chelating capacity to form nontoxic complexes with HM substrates in the rhizosphere to reduce HM uptake and accumulation in the plants, further limiting phytotoxicity [7,95]. 

### 3.2. Applied Metabolomics in the Elucidation of Plant Response to Osmotic Stress

Osmotic stress is known to occur under all different environmental stress conditions, including drought, salinity, cold/freezing, or high-temperature conditions, and heavy metal toxicity and its tolerance play a crucial role in plant survival through stress conditions [96,97]. Due to this commonality, metabolomics studies dedicated to evaluating osmotic stress response in plants are minimal, with a mention of osmoregulation as a side effect of the stress conditions mentioned above, particularly in studies concerned with drought and salinity stress. Plants growing under saline and drought conditions encounter osmotic stress due to disrupted ion homeostasis and imbalance, which leads to disturbances in various metabolic and physiological processes that subsequently affect plant growth and development [98]. Plants have implemented controlled up-and-down-regulation of primary and specialised secondary metabolites to constitute a defence mechanism for osmotic stress tolerance. Analysing these stress-responsive metabolic features could be essential in assessing the stress adaptation strategies of plants.

Metabolomics studies and the reviewed literature have reported on perturbation in primary-amino acids, sugars and organic acids and derivatives, and secondary metabolic profiles inclusive of phenolics acids, flavonoids, and phytohormones in plants responding to osmotic stress, associated drought, salinity, and HM toxicity [61,65,66,67]. A time-course comparative metabolic profiling of osmotic stress-tolerant and sensitive hulless barley (*Hordeum vulgare* L. *var. nudum* Hook. f.) revealed differential metabolite reprogramming in the osmotic stress-tolerant (XL) and susceptible (D) barley varieties [99]. A Q TRAP LC-ESI-MS/MS analysis showed a total of 22 diverse compounds from the classes of flavonoids, glycerophospholipids, and amino acid derivatives that were differentially regulated in the XL metabolome responsive to osmotic stress. The high accumulation of flavonoids was attributed to their osmotic-stress-induced ROS scavenging capabilities. The onset of osmotic stress strongly induced phosphatidylcholines; these are compounds of significant glycine precursors betaine (GB), an essential organic osmolyte that accumulates in plants responding to osmotic stress caused by salinity, drought, extreme temperatures, and HM stress [99,100]. 

Capillary electrophoresis time-of-flight mass spectrometry (CE-TOF-MS) analysis was used to investigate the metabolic profiles of two rice (*Oryza sativa* L.) cultivars resistant (IR 58) and sensitive (Basilanon) to osmotic stress [101]. IR 58 showed an increased accumulation of stress-induced metabolites involved in sugar metabolism (sucrose 6′-phosphate, glucose 1-phosphate), polyamine, and phenylpropanoid metabolisms (spermine, spermidine, gamma-aminobutyric acid (GABA)), and glutathione metabolism (glutathione, cysteine, cadaverine). Amino acids such as proline, serine, glutamine, and asparagine were up-regulated in the roots of the resistant cultivar. Moreover, the authors reported increased tricarboxylic acid (TCA) cycle intermediates such as citric acid, cis-Aconitic acid, isocitric acid, fumaric acid, and malic acid under osmotic stress compared to the control. Reportedly, a cumulative increase of these metabolites led to improved biological functions such as energy production and antioxidant defence under osmotic stress [101]. Another study found that response to osmotic stress and oxidative damage are vital processes underlying NaCl tolerance [102]. A targeted LC-MS/MS analysis revealed differential regulation of metabolite in halophytes species, a positive correlation between the content of the osmolyte proline, and salinity treatment in roots and leaves of *Sesuvium portulacastrum*. In contrast, other osmolytes, such as glycine betaine and polyols, were up-regulated in *Salicornia brachiate* and *Suaeda maritima*. Benjamin and colleagues [102] further used UHPLC/QTOF-MS to analyse metabolic profiles of the three halophytes, which allowed annotation of 3600 compounds, including flavonoids and related metabolites, phenylpropanoids and lignans, alkaloids and amine compounds, terpenes, lipids, hormones, and carbohydrates which increased in accumulation in response to salt treatments through osmotic adjustment and mitigation of oxidative damage.

## 4. Conclusions and Perspectives

The successful implementation of metabolomics techniques has enriched our knowledge of the biochemical landscape of plants; significant progress has been made in the elucidation of the metabolic state of plants under specified environmental conditions, including biotic and abiotic stress. These techniques and related computational bio/cheminformatics tools have allowed an in-depth evaluation of the plant’s cellular homeostasis, metabolic reprogramming, and the adaptive mechanisms plants use in response to environmental stimuli. To date, metabolomics-guided metabolic marker identification carries great potential for drawing inferences on the impact of stress conditions on plants. Such metabolic markers as sugars, amino acids, organic acids, and carbohydrates, as well as some specialised secondary metabolites, including flavonoids and related phenylpropanoids, have been reported to mediate plant stress response through their antioxidant properties, ROS scavenging, and metal chelating to mitigate HM toxicity; additionally, these metabolites assist in osmotic adjustment and the maintenance of homeostasis in the plant for tolerance against abiotic stress. Advanced applications of metabolomics techniques, aided by technological improvements including machine learning and computational tools displaying rapid compound dereplication, predictive capabilities, and a library of metabolite databases able to provide rapid metabolite annotation, can help advance comprehensive large-scale exploration of the plant metabolome.

Furthermore, these advances can be expected to continue, given the current and expected developments of 4IR technologies in plant metabolomics, automated and standardised workflows, applied robotics systems in sample preparation, remote data acquisition, and big data cloud storage/sharing. The development of handheld (portable) analytical devices is also expected to fast-track plant metabolomics by on-field tissue analysis without the requirements of extraction and sample preparatory methods, allowing for raw data acquisition and real-time data analysis to reveal the metabolic and cellular status of the plant. This approach will thus allow for the rapid development of methods for application in crop improvement, particularly in crop productivity, yield, and resilience against abiotic stress.

## Data Availability

Data sharing not applicable.
